# Implications of Increased Access to Buprenorphine for Medical Providers in Rural Areas: A Review of the Literature and Future Directions

**DOI:** 10.7759/cureus.19870

**Published:** 2021-11-24

**Authors:** Hannah M Gregory, Veronica M Hill, Robert W Parker

**Affiliations:** 1 Anesthesiology and Critical Care, Alabama College of Osteopathic Medicine, Dothan, USA; 2 Family Medicine, Alabama College of Osteopathic Medicine, Dothan, USA; 3 Pharmacology, Alabama College of Osteopathic Medicine, Dothan, USA

**Keywords:** rural health, opioid medication, opioid agonist, opioid use disorder (oud), medication assisted treatment (mat), medications for opioid use disorder (moud), suboxone, naloxone, dea waiver, buprenorphine

## Abstract

Buprenorphine/Naloxone (Suboxone®) is an efficacious treatment for opioid use disorder (OUD) due to its more convenient dosing, superior safety profile, and decreased incidence of negative side effects when compared to other forms of medications for opioid use disorder (MOUD). In the United States, updated legislation in 2021 entitled, "The Practice Guidelines for the Administration of Buprenorphine for Treating Opioid Use Disorder",* *released by the Department of Health and Human Services, creates an exemption for the previously required Drug Addiction Treatment Act of 2000 (DATA) waiver for buprenorphine prescribing for clinicians. This legislation was born out of a need for making MOUD more accessible for patients living with OUD as rates of opioid-related deaths in the United States have continued to rise and have increased disproportionately during the time period of the COVID-19 pandemic. This legislation has the potential to improve access to MOUD across all geographic locations, but may have the most profound impact in rural areas where significant disparities and challenges still exist in patients’ ability to access buprenorphine. The purpose of this literature review is to 1) examine how MOUD prescribing has changed after previous legislation changes, 2) explore the current state of buprenorphine access for treatment of OUD in rural America, 3) detail existing barriers in patients' ability to access MOUD, and 4) discuss future directions and considerations as a result of new legislation. This literature review found several existing barriers to receiving MOUD such as increasing costs, insufficient education, significant stigma, and the need for more innovative methods of delivery. We also found that there is currently a large opportunity for growth in the number of rural clinicians able to prescribe buprenorphine, particularly in primary care, that may now occur as a result of this new legislation. Overall, this legislation has the potential to have a positive impact on combating OUD, especially in rural areas, and may be a critical step towards ending the current opioid epidemic in the United States as these described barriers are addressed.

## Introduction and background

Pharmacology of buprenorphine and naloxone

Medications for opioid use disorder (MOUD), formally referred to as medication assisted treatment (MAT), are medications that can be prescribed to patients who want to stop using opioids [1]. The older terminology “medication-assisted treatment” is being phased out since it implies that medications play a secondary or supporting role to other treatment forms for opioid use disorder (OUD). The new terminology, “medications for opioid use disorder”, defines medication as the primary method of treatment for OUD that can stand alone as its own treatment form. Opioid agonists (i.e., buprenorphine, methadone) and opioid antagonists (i.e., naltrexone) are all medications that can be used as forms of MOUD [2]. Buprenorphine’s desirable clinical effects and safety profile contribute to its status as one of the recommended MOUD [2].

Buprenorphine/Naloxone (Suboxone®) is a combination medication administered for treatment and management of opioid use disorder (OUD). Buprenorphine is a partial mu-opioid receptor agonist effective in maintenance pharmacotherapy of OUD due to its long half-life, greater safety in overdose compared to full mu-opioid receptor agonists, and decreased incidence of opioid-induced side effects like respiratory depression, constipation, and tolerance [3-5]. Buprenorphine is unique from other opioids as it is a partial but extremely potent agonist at the mu-opioid receptor. Its high affinity and low intrinsic activity at the receptor allow buprenorphine to displace other full mu-opioid agonists, leading to more desirable clinical properties such as lower potential for abuse, lower levels of physical dependence, and lower incidence of withdrawals if a dose is missed [3]. Additionally, its slow dissociation from the mu-opioid receptor allows for its prolonged therapeutic effects and high efficacy in treating OUD as well as pain [3].

When used to treat OUD, buprenorphine is administered with naloxone to lower its abuse potential [2]. Naloxone is an opioid antagonist most commonly used to rapidly reverse opioid overdose. When administered with buprenorphine, it is given via a film or tablet that dissolves sublingually [2]. Lower amounts of naloxone are absorbed sublingually compared to other routes of administration, allowing buprenorphine to have its desired effects without naloxone competition at the opioid receptor [2]. If naloxone is instead injected into the bloodstream, it will precipitate undesirable opioid withdrawal symptoms (i.e., irritability, anxiety, nausea, vomiting, diarrhea, muscle aches, excessive sweating, etc.) in someone who is opioid-dependent [6]. The addition of naloxone to buprenorphine discourages people who are dependent on intravenous opioids from attempting to inject buprenorphine due to its ability to activate opioid receptors and cause opioid-induced euphoria [6]. Buprenorphine/naloxone is a desirable choice of drug for the treatment of OUD when compared to other drugs due to its increased safety profile, lower potential for relapse, more convenient dosing, ability to manage opioid cravings while helping lower dependency gradually, and ability to start relatively soon after the patient stops taking other opioids [2,3,7]. 

Previous requirements for buprenorphine prescribing

Maintenance pharmacotherapy of buprenorphine/naloxone does not require daily clinic visits, making it desirable for patients who are unable to visit a clinic daily for treatment of OUD. However, a major hurdle for patients in the United States to access buprenorphine/naloxone has been the requirement for clinicians to obtain a Drug Addiction Treatment Act of 2000 (DATA) waiver from the Drug Enforcement Administration (DEA) to prescribe buprenorphine. To apply for the DATA waiver, a physician must have either taken an eight-hour training course to qualify or possessed the appropriate board certification. Nurse practitioners, physician assistants, certified nurse specialists, certified registered nurse anesthetists, and certified nurse-midwives were required to complete 24 hours of training to be eligible to apply for a DATA waiver for buprenorphine prescription privileges [8]. After completing the appropriate training and obtaining a DATA waiver for prescription of schedule III, IV, or V narcotic drugs, subsequent prescriptions of qualifying drugs for the treatment of OUD must include both the DEA registration number and the clinician’s identification number (“X number”) [9].

Opioid use disorder in rural communities and COVID-19 implications

The United States spends over 100 billion dollars a year on costs related to pain management and opioid dependence [10]. Even more troubling, the Center for Disease Control and Prevention (CDC) reports that deaths due to opioid overdose continue to increase steadily every year [11]. The CDC estimates that nearly 50,000 people die each year from an opioid overdose [12] and deaths due to opioid use continue to grow faster in rural areas than in metropolitan areas [13]. Rural areas may be disproportionately affected by OUD due to geographical isolation, an insufficient number of local providers able to manage MOUD, increased ability to manufacture illicit drugs in rural agriculture industries, lower-income households, lack of long-term supportive therapies available, decreased prevalence of law enforcement, and increased prevalence of concurrent abuse of other substances, specifically alcohol [14,15]. Overdose deaths have surged since the emergence of the COVID-19 pandemic, possibly due to a variety of reasons, including lack of access to medical treatment, social isolation, and increased mental health distress [16]. From June 2019 to May 2020, the highest number of overdose deaths in 12 months ever was recorded. Over 81,000 people died of drug overdoses in the United States during this time, a record-breaking number that CDC officials suggested is related to the COVID-19 pandemic [16,17]. 

New legislation for buprenorphine prescribing

The burden of increasing opioid drug overdoses in recent years, which has only been bolstered by the COVID-19 pandemic, highlighted the need for legislation that would lessen the restrictions on buprenorphine prescribing for clinicians to treat OUD and increase their ability to reach more patients. On January 14, 2021, the United States Department of Health and Human Services announced a plan to update its guidelines and create an exemption for the required DATA waiver that was previously needed for clinicians to prescribe buprenorphine for the management of OUD. The official guidelines were published on April 28, 2021, and state that:

“The Practice Guidelines for the Administration of Buprenorphine for Treating Opioid Use Disorder provides eligible physicians, physician assistants, nurse practitioners, clinical nurse specialists, certified registered nurse anesthetists, and certified nurse midwives, who are state-licensed and registered by the DEA to prescribe controlled substances, an exemption from certain statutory certification requirements related to training, counseling and other ancillary services.” [18].

This new and current legislation allows for expanded prescribing access to buprenorphine for the treatment of OUD for up to 30 patients per provider. Under these guidelines, providers no longer have to partake in a qualifying DATA waiver training course to be able to prescribe buprenorphine. The purpose of this literature review is to discuss the current state of buprenorphine access and utilization for treatment of OUD in rural America and future directions and considerations as a result of new legislation.

## Review

Methods

**Figure 1:**
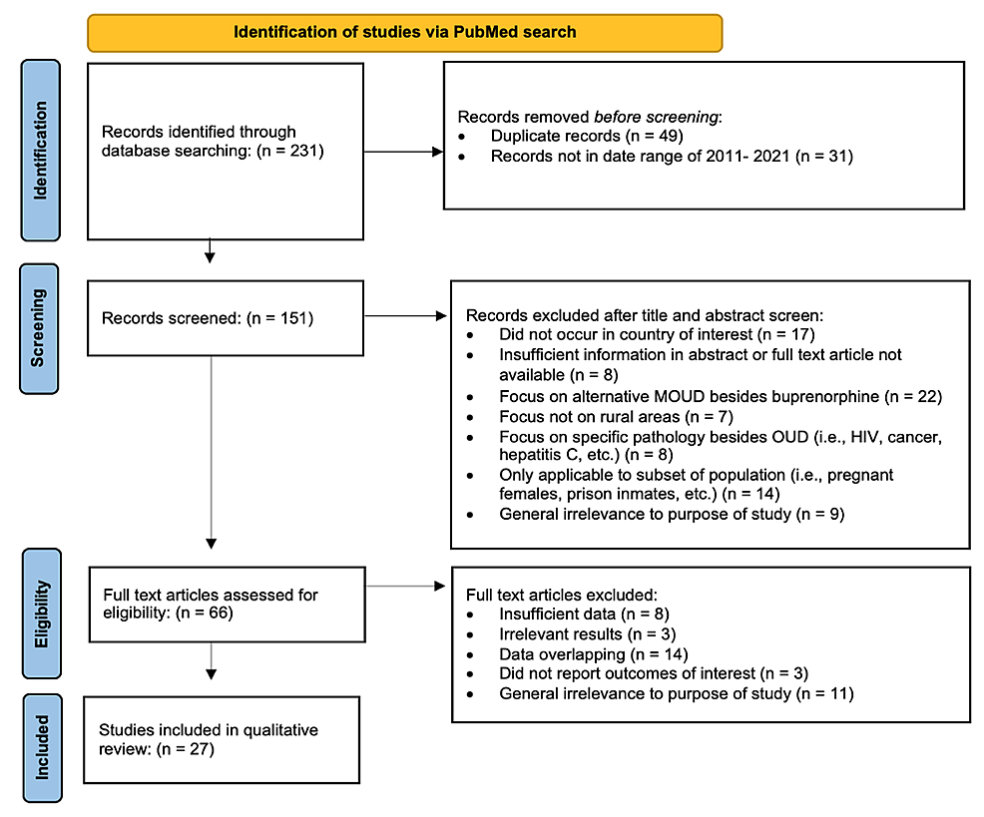
PRISMA 2020 flow diagram for new systematic reviews showing process of identifying studies after applying inclusion and exclusion criteria.

A review of the literature was performed using preferred reporting items for systematic reviews and meta-analyses (PRISMA) guidelines. A search on PubMed and PubMed Central was conducted, including keywords like “DEA waiver geographic distribution,” “opioid use disorder in rural areas,” and “Buprenorphine use in rural areas”. Studies were limited within the last 10 years from 2011 to 2021, with no restriction on the age of participant or type of study or literature. Study locations were limited to within the United States. Literature that could not be directly related or applied to rural areas was excluded. Additionally, 1) literature that discussed other forms of MOUD besides buprenorphine, 2) literature that only focused on a subset of the population (i.e., pregnant females, prison inmates, etc.) with OUD, and 3) literature with a primary focus on substance abuse-related pathologies other than OUD (i.e., HIV, cancer, hepatitis C) were excluded. Literature was only included that 1) focused on buprenorphine as a form of MOUD, or 2) focused on OUD and MOUD in rural areas. While critically reviewing all literature, the aim was to identify well-documented trends and changes in buprenorphine access in rural areas as well as areas of opportunity, potential barriers, and recommendations for proceeding with buprenorphine prescribing under the new legislation. Particular attention was given to trends in buprenorphine access and prescribing after past legislation changes. This information was used to form predictions on changes and barriers that may occur or persist as a result of new legislation. Initially, 231 PubMed studies were identified. After applying inclusion and exclusion criteria, the number of appropriate studies was narrowed down to 27 for qualitative review. The process of identifying and excluding articles can be seen in the PRISMA flow diagram (Figure 1). The titles of all articles included in the final qualitative review and the primary support they provided for this literature review can be seen in Table 1. 

**Table 1:** Chart of all articles (27) included in qualitative review.

Author	Title	Primary Support Used for Literature Review
Andrilla CHA, Patterson DG	Tracking the geographic distribution and growth of clinicians with a DEA waiver to prescribe buprenorphine to treat opioid use disorder	The number of DEA-waivered clinicians has improved across all geographic categories, but rural communities still experience significant treatment disparities and many still lack providers.
Rosenblatt RA, Andrilla CH, Catlin M, Larson EH	Geographic and specialty distribution of US physicians trained to treat opioid use disorder	Psychiatry is the specialty with the highest rate of DEA-waivered physicians, although primary care most often serves rural patients. Many counties lack providers altogether.
Amiri S, Hirchak K, McDonell MG, Denney JT, Buchwald D, Amram O	Access to medication-assisted treatment in the United States: Comparison of travel time to opioid treatment programs and office-based buprenorphine treatment	Compared to urban residents, rural residents have longer travel times to access treatment for OUD.
Klein TA, Geddes J, Hartung D	The Geographic Impact of Buprenorphine Expansion to Nurse Practitioner Prescribers in Oregon	The contribution of NPs had a particularly large impact on treating patients with OUD in rural areas, where they provided over 1/3rd of all buprenorphine prescriptions by the end of 2018.
Barnett ML, Lee D, Frank RG	In Rural Areas, Buprenorphine Waiver Adoption Since 2017 Driven By Nurse Practitioners And Physician Assistants	From 2016-2019 the number of DEA-waivered clinicians in rural areas increased by 111%. NPs and PAs accounted for more than half of this increase and were the first waivered clinicians in 285 rural counties.
Andrilla CHA, Patterson DG, Moore TE, Coulthard C, Larson EH	Projected Contributions of Nurse Practitioners and Physicians Assistant to Buprenorphine Treatment Services for Opioid Use Disorder in Rural Areas	NPs and PAs are projected to increase the number of rural patients treated with buprenorphine by 10,777 (15.2%) and have considerable potential to reduce rural disparities regarding access to MOUD.
Pham S, Haigh A, Barrett E	Statewide Availability of Buprenorphine/Naloxone in Acute Care Hospitals	In one predominately rural state with an increased opioid overdose death rate, approximately 1/2 of acute care hospitals did not have buprenorphine/naloxone available to patients. Efforts to increase buprenorphine/naloxone availability in rural hospitals are needed.
Jones EB	Medication-Assisted Opioid Treatment Prescribers in Federally Qualified Health Centers: Capacity Lags in Rural Areas	Health centers in rural areas had lower odds of providing on-site buprenorphine treatment and had lower odds of expressing interest in expanding the availability of buprenorphine treatment.
Bond Edmond M, Aletraris L, Roman PM	Rural substance use treatment centers in the United States: an assessment of treatment quality by location	Rural treatment centers have access to fewer resources to treat OUD with. They are less likely to offer buprenorphine for MOUD but are more likely to treat adolescents with specialized treatment.
Quest TL, Merrill JO, Roll J, Saxon AJ, Rosenblatt RA	Buprenorphine therapy for opioid addiction in rural Washington: the experience of the early adopters	Buprenorphine was reported to be efficacious in the treatment of OUD by all rural physician respondents but major barriers exist that limit access to treatment.
Cochran G, Cole ES, Warwick J, Donohue JM, Gordon AJ, Gellad WF, Bear T, Kelley D, DiDomenico E, Pringle J	Rural access to MAT in Pennsylvania (RAMP): a hybrid implementation study protocol for medication assisted treatment adoption among rural primary care providers	Medicaid provides health coverage to 24% of non-elderly adults in rural areas and nearly 40% of adults with OUD in the US. Medicaid Managed Care Organizations (MCOs) may be utilized to sustain MOUD expansion within the clinic.
McClellan C, Fingar KR, Ali MM, Olesiuk WJ, Mutter R, Gibson TB	Price elasticity of demand for buprenorphine/naloxone prescriptions	A doubling in the price of buprenorphine/naloxone disproportionately affects rural patients and should be considered before insurers increase out-of-pocket costs.
Sigmon SC	Innovations in efforts to expand treatment for opioid use disorder	Use of sustained release buprenorphine can reduce risk for overdose, morbidity and mortality, while rural patients are waiting for more comprehensive treatment.
Andrilla CHA, Moore TE, Patterson DG	Overcoming Barriers to Prescribing Buprenorphine for the Treatment of Opioid Use Disorder: Recommendations from Rural Physicians	Providers report many barriers to utilizing their DEA-waiver, including medication diversion and stigma.
Richard EL, Schalkoff CA, Piscalko HM, Brook DL, Sibley AL, Lancaster KE, Miller WC, Go VF	"You are not clean until you're not on anything": Perceptions of medication-assisted treatment in rural Appalachia	Stigma surrounding MOUD needs to be addressed in order to tackle the opioid epidemic, especially in rural areas.
DeFlavio JR, Rolin SA, Nordstrom BR, Kazal LA Jr.	Analysis of barriers to adoption of buprenorphine maintenance therapy by family physicians	Many family medicine physicians feel they regularly see patients addicted to opioids but there are several logistical barriers that must be addressed in order to offer in primary care settings.
Sorrell TR, Weber M, Alvarez A, Beste N, Hollins U, Amura CR, Cook PF	From policy to practice: Pilot program increases access to medication for opioid use disorder in rural Colorado	Efforts are needed to better serve patients in rural counties that are severely affected by the opioid crisis, including a reduction of stigma, increased community coordination, and adequate reimbursement.
Franz B, Dhanani LY, Miller WC	Rural-Urban Differences in Physician Bias Toward Patients With Opioid Use Disorder	Physicians in rural areas reported higher levels of bias toward patients with OUD than their urban counterparts and may benefit from bias reduction interventions.
Cole TO, Robinson D, Kelley-Freeman A, Gandhi D, Greenblatt AD, Weintraub E, Belcher AM	Patient Satisfaction With Medications for Opioid Use Disorder Treatment via Telemedicine: Brief Literature Review and Development of a New Assessment	Telemedicine is being increasingly used to treat patients with OUD and may have particular value in rural areas. Treating OUD with telemedicine produces clinical outcomes similar to face-to-face interactions.
Weintraub E, Greenblatt AD, Chang J, Himelhoch S, Welsh C	Expanding access to buprenorphine treatment in rural areas with the use of telemedicine	Telemedicine is a potential tool to expand MOUD to underserved rural populations. Patients with OUD can maintain opioid-free status for months after beginning treatment with MOUD via telemedicine.
Huskamp HA, Busch AB, Souza J, Uscher-Pines L, Rose S, Wilcock A, Landon BE, Mehrotra A	How Is Telemedicine Being Used In Opioid And Other Substance Use Disorder Treatment?	Several different models for telemedicine may be used for treatment of OUD. However, telemedicine may be insufficient for treatment of some patients with OUD.
Oleskowicz TN, Ochalek TA, Peck KR, Badger GJ, Sigmon SC	Within-subject evaluation of interim buprenorphine treatment during waitlist delays	Interim treatment with buprenorphine may be suitable for patients in rural areas where there are limited treatment options as it can reduce opioid-associated risks.
Magidson JF, Jack HE, Regenauer KS, Myers B	Applying lessons from task sharing in global mental health to the opioid crisis	There is a severe shortage of trained clinicians to meet the needs of patients with OUD. This may be improved by implementation of lay healthcare workers and task sharing into clinic settings.
Look KA, Kile M, Morgan K, Roberts A	Community pharmacies as access points for addiction treatment	Community pharmacies are an integral part of the rural health care system and could be used to support OUD treatment in counties with limited access to formal treatment facilities.
Cochran GT, Engel RJ, Hruschak VJ, Tarter RE	Prescription Opioid Misuse Among Rural Community Pharmacy Patients: Pilot Study for Screening and Implications for Future Practice and Research	Community pharmacies have great presence and reach in rural areas. Pharmacist-led medication adherence interventions could benefit patients in rural communities with OUD.
Deyo-Svendsen M, Cabrera Svendsen M, Walker J, Hodges A, Oldfather R, Mansukhani MP	Medication-Assisted Treatment for Opioid Use Disorder in a Rural Family Medicine Practice	Suboxone is the most effective and practical option for incorporating MOUD into a primary care outpatient family medicine practice and can decrease the risk of other disease.
Fried JE, Basu S, Phillips RS, Landon BE	Financing Buprenorphine Treatment in Primary Care: A Microsimulation Model	In the current fee-for-service healthcare workplace environment, offering office-based therapy for OUD with buprenorphine can be a financially beneficial choice for rural primary care practices.

Current state, changes, and challenges of buprenorphine prescribing in rural areas

Despite overall increases in recent years in the number of providers able to prescribe buprenorphine, a large disparity still exists for patients in rural (versus urban) areas in their ability to access buprenorphine [19-21]. This overall increase in DEA-waivered clinicians was largely due to the Comprehensive Addiction and Recovery Act (CARA - 2016) that permitted nurse practitioners and physician assistants (NPs and PAs) to prescribe buprenorphine with a DEA waiver [19]. As a result of this legislation, the number of DEA-waivered clinicians more than doubled between December 2017 and July 2020 from 37,869 to 98,344 [19]. Even with overall increases across all geographic categories, more than half of all small and remote rural counties still lacked a provider able to prescribe buprenorphine, representative of significant geographical disparity for patients living in rural areas with OUD [19]. Before CARA, more than 30 million Americans were living in counties without access to buprenorphine treatment [20]. Only 2.2% of the US physician workforce had obtained waivers to prescribe buprenorphine, according to the official DEA DATA waiver list [20]. Of the physicians who were waivered, 90.4% of them were practicing in urban counties, creating a large disparity in the ability of patients with OUD in rural areas to receive MOUD [20]. As of 2021, there has been a significant overall improvement in the ability to access buprenorphine treatment, but challenges still exist for rural patients, such as significantly longer drive times to clinics where they can receive MOUD [21]. However, when compared to utilization of methadone for MOUD, buprenorphine was relatively more accessible for rural patients as determined by shorter drive times to the nearest treatment site [21]. This suggests that buprenorphine may be more feasible or efficacious than other forms of MOUD for rural patients. Its documented ability to be more successfully and conveniently accessed in rural areas should be built upon and be used to encourage rural providers to continue the expansion of buprenorphine as a primary form of MOUD. Even though there are still disparities for rural patients, there is evidence that improved legislation has a substantial impact on the number of patients able to receive buprenorphine for OUD. This suggests that new 2021 legislation may have a particularly positive impact in rural areas as there is a large opportunity for growth in the number of rural clinicians able to prescribe buprenorphine.

After NPs and PAs were granted eligibility to prescribe buprenorphine, they have consistently been key players in efforts to increase buprenorphine access for rural patients [22-24]. A study from one state suggests that the total number of buprenorphine prescriptions increased in rural areas immediately after CARA implementation [22]. NPs and PAs were the first waivered clinicians in 285 rural counties, expanding access to buprenorphine for over 5.7 million residents and increasing the total buprenorphine treatment capacity by 90% [23]. As recent as 2020, NPs and PAs were projected to increase the number of rural patients treated with buprenorphine by 10,777 (15.2%) [24]. With 2021 legislation that eases buprenorphine restrictions for several clinicians, we project that NPs and PAs will continue to have a significant impact due to their action after previous legislation changes and because NPs are often more likely to treat rural patient populations than physicians are [25].

Among all medical specialties, physician psychiatrists have previously held the majority of DEA waivers for buprenorphine prescribing [20]. This is indicative of a large area for potential growth with new legislation as primary care physicians (namely family practice and internal medicine) are the major health providers in rural America and may be more likely than psychiatrists to treat rural patients with OUD [20]. Additionally, physicians aged 35 years old and younger represented only 2.6% of the total buprenorphine prescribers [20]. We believe these findings represent a large opportunity for increased education surrounding buprenorphine prescribing for MOUD in medical education and residency training so young physicians will feel more confident in their ability to manage MOUD when they enter the physician workforce.

Data suggests that though buprenorphine may be more accessible than other forms of MOUD for rural patients [21], there may still be an overall lack of access to buprenorphine in rural hospitals, substance use treatment centers, and community health centers [26-28]. Health centers in rural areas had lower odds of providing on-site buprenorphine treatment and expressed interest in expanding the availability of buprenorphine treatment [26]. Additionally, a study conducted in one rural state with increased rates of opioid overdose deaths compared to the national average showed that almost ½ of their acute care hospitals did not have buprenorphine/naloxone available to patients receiving inpatient treatment [27]. One important thing to note is that although rural health centers have decreased access to resources and medications (particularly buprenorphine) for treatment of OUD, they were more likely than urban health centers to provide specialized treatment for adolescents [28]. This only underscores the importance of the new legislation that eases restrictions on buprenorphine prescribing and access, especially in rural areas where other forms of MOUD are less available and there are vulnerable populations (i.e., adolescents) being treated more frequently.

One of the biggest challenges to date with increased buprenorphine prescribing for OUD remains financial concerns over insurance coverage, increased costs, and Medicaid reimbursement [29-31]. Evidence shows that increases in the price of buprenorphine/naloxone have the most pronounced negative effects on rural patients and patients from the south [29]. When the price of buprenorphine/suboxone increases, these geographic groups are the least likely among all others to refill their prescription [29]. As aforementioned, rural patients deal with unique and complex circumstances that must not be complicated by barriers to medication adherence, such as rising costs. Additionally, concerns over Medicaid coverage continue to rise. Medicaid is the largest source of third-party coverage for rural patients with OUD and provides coverage for nearly 40% of patients with OUD in the United States [30,31]. Physicians who prescribe buprenorphine for the treatment of OUD report that they often had difficulty in getting patients who were covered by Medicaid approved for reimbursement. Even in cases where buprenorphine was covered, it was relatively rare for Medicaid to extend the coverage beyond six months and never beyond 12 months [30]. This means that although access to buprenorphine may be expanding, many patients who are in need may not be able to access it or achieve sustained treatment due to a lack of funding and coverage. Along with this new legislation, improved insurance compensation must occur, and insurers should be encouraged to cover MOUD costs for longer periods and be discouraged from increasing out-of-pocket costs. Increased financial support from federal Medicaid and state-level Medicaid Managed Care Organizations (MCOs) should be pursued as it can improve access to MOUD for several rural patients and help them achieve and sustain sobriety [31]. In addition to this major financial barrier, other challenges shared by rural physicians are lack of a physician support network, lack of adequate clinic staff who were trained on caring for patients with OUD, a need to address mental health concerns through additional counseling, time restraints, legal regulations, stigma, and concerns over buprenorphine diversion [30,32-35].

Future directions as a result of new legislation

With new legislation in 2021, changes in buprenorphine prescribing should be considered as it becomes more accessible for patients across all geographic domains. One of the most important things to do as part of the new legislation is to reduce the stigma surrounding the use of buprenorphine to treat OUD and the stigma surrounding providers who prescribe MOUD. Stigma towards MOUD is still commonly present among healthcare professionals, law enforcement workers, judicial officials, and other community members [34,36]. Clinicians in rural areas were found to report increased levels of bias toward patients with OUD than their urban counterparts [37]. MOUD-related stigma will need to be addressed to fully tackle the opioid epidemic, especially in rural areas. Clinicians in rural areas may consider bias reduction interventions as these efforts may have the greatest impact on improving access to MOUD in these areas [34,37].

Other future directions should consider changes in how buprenorphine is delivered for MOUD. The utilization of both telemedicine [38-40] and interim-dosing buprenorphine [32,41] have been suggested to be efficacious delivery methods for MOUD in rural areas. Treating OUD via telemedicine has been shown to produce clinical outcomes similar to in-person interactions [38,39,42]. Utilizing telemedicine to treat and monitor patients who are taking buprenorphine/naloxone for OUD may have particular value in rural areas where there is a shortage of healthcare providers, and patients may need to drive long distances to access a provider. Treatment with buprenorphine is effectively delivered via telemedicine to patients with OUD in rural drug treatment programs [39]. One study demonstrated that when rural patients remain engaged in telemedicine treatment, the large majority maintain opioid-negative urine toxicology when tracked over multiple months [39]. Multiple models exist for monitoring treatment of OUD with buprenorphine via telemedicine, including a hybrid of virtual and in-person visits, which combines patient counseling into the treatment plan [40]. Even though telemedicine has shown promise in rural areas, it does not come without barriers and considerations. The Ryan Haight Online Pharmacy Consumer Protection Act of 2008 was created to regulate online medication prescribing in response to the tragic death of a teenager who died from overdosing on an opioid he obtained online without meeting with a clinician [40,43]. The act required any clinician issuing a prescription for a controlled substance to meet with the patient face-to-face and conduct an in-person medical evaluation before doing so (with some specific exemptions) [43]. More recent legislation entitled the Substance Use-Disorder Prevention that Promotes Opioid Recovery and Treatment (SUPPORT) for Patients and Communities Act was signed on October 24, 2018, to lessen the restrictions on telemedicine prescribing [44]. The SUPPORT Act allows telemedicine providers to register with the DEA and prescribe controlled substances (such as buprenorphine) without an in-person exam first [40,44]. Telemedicine prescribing has the potential to help clinicians who recently gained the ability to prescribe buprenorphine under 2021 legislation to reach more rural patients with OUD. However, the pros and cons of telemedicine should always be considered to meet the individualized needs of each patient as it is not a one-size-fits-all approach and may not be the best therapy option for some patients. Another future direction for changes in how buprenorphine is delivered for MOUD is the utilization of interim-dosing buprenorphine. As more clinicians are becoming eligible to prescribe buprenorphine under 2021 legislation, interim-dosing buprenorphine may be considered as it can bridge temporary delays between treatment and is associated with decreased morbidity and mortality [32]. Studies suggest that interim dosing with buprenorphine is associated with a statistically significant reduction in the use of illicit opioids and intravenous drugs while a patient is on the waiting list to receive comprehensive treatment [32]. Interim-dosing buprenorphine can reduce opioid-related risks and may be suitable for patients in rural areas where there are limited treatment options or the wait times to receive treatment are significant [41].

Other future directions should include the implementation of more education on MOUD and the prescribing of buprenorphine into NP and PA education programs as both professions have demonstrated the ability to have a substantial impact on the treatment of patients, particularly in rural areas, with OUD. Medical schools and residency programs should provide educational opportunities for student physicians and residents to learn about MOUD. If education on MOUD becomes more prevalent in medical education, we predict there will be a significant increase in the number of young and newly graduated physicians who feel confident and well-informed on how to treat patients addicted to opioids in their own areas of practice. Additionally, there must be increased support and resources for clinicians in rural areas who prescribe buprenorphine for MOUD. This may be done through the creation of a clinician collaboration network for experienced buprenorphine prescribers to share insight and education with new prescribers [30], increased education and staff support for primary care clinicians [35], implementation of lay healthcare workers, and task sharing into treatment plans [45], and utilization of community pharmacies as a point of access to care in rural communities as an added layer of support [46,47]. We also suggest the consideration of incentives that will increase the level of provider participation, including but not limited to decreased prescriber regulations (i.e., prior authorization requirements) and increased reimbursements for services provided. Analyzing the literature for growth opportunities suggest that implementation of these changes along with new important legislation may lead to improved clinician satisfaction [30,33], improved overall rural community health [48], increased utilization of primary care services [48], and increased revenue that can help sustain rural healthcare offices [49].

## Conclusions

As the opioid epidemic continues to affect the United States and has a disproportionate effect on rural areas, it is critical for legislation to evolve to meet the needs of clinicians who treat OUD and their patients. New legislation in 2021 that aims to ease the restrictions on buprenorphine prescribing will improve access to MOUD across all geographic locations but may have the most profound impact in rural areas where significant disparities exist. Barriers to receiving MOUD in rural areas such as long commute times, lack of access to buprenorphine in rural hospitals and community health centers, lack of primary care physicians who manage MOUD, rising costs, issues with Medicaid reimbursement, concerns over buprenorphine diversion, utilization of innovative approaches to buprenorphine prescribing, and significant stigma are profound and must be considered and addressed as a result of new legislation. Support for clinicians under this new legislation can be attained through the formation of collaboration networks, increasing educational awareness surrounding MOUD for staff and students, implementation of lay healthcare workers and task sharing into treatment plans, utilization of community pharmacies as a point of access to care, and ensuring local pharmacies stock both buprenorphine/naloxone and naloxone. This legislation has the potential to significantly improve the health and quality of life for many people living in the United States with OUD. We anticipate there will be lower rates of relapse, decreased incidence of risky opioid-related behaviors, and improved rates of opioid-related morbidities and mortality as the reach of treatment to patients with OUD will be increased substantially. Future studies should track and quantify the impact this new legislation has, particularly among rural and vulnerable populations, and detail what methods are most successful for implementation of MOUD with buprenorphine into clinics, hospitals, pharmacies, etc. Existing barriers and newly arising barriers should also be detailed so legislation and education can continue to evolve until the opioid epidemic has been eradicated.
